# Anxiolytic effects of NLRP3 inflammasome inhibition in a model of chronic sleep deprivation

**DOI:** 10.1038/s41398-020-01189-3

**Published:** 2021-01-14

**Authors:** Chad Smith, Kyle J. Trageser, Henry Wu, Francis J. Herman, Umar Haris Iqbal, Maria Sebastian-Valverde, Tal Frolinger, Emma Zeng, Giulio Maria Pasinetti

**Affiliations:** 1 Department of Neurology, Icahn School of Medicine at Mount Sinai, New York, New York 10029 USA; 2 Geriatric Research, Education and Clinical Center, James J. Peters Veterans Affairs Medical Center, Bronx, NY 10468 USA

**Keywords:** Molecular neuroscience, Neuroscience

## Abstract

Sleep deprivation is a form of stress that provokes both inflammatory responses and neuropsychiatric disorders. Because persistent inflammation is implicated as a physiological process in anxiety disorders, we investigated the contributions of NLRP3 inflammasome signaling to anxiety and anxiolytic properties of flavanol diets in a model of chronic sleep deprivation. The results show a flavanol-rich dietary preparation (FDP) exhibits anxiolytic properties by attenuating markers of neuroimmune activation, which included IL-1β upregulation, NLRP3 signaling, and microglia activation in the cortex and hippocampus of sleep-deprived mice. Production of IL-1β and NLRP3 were critical for both anxiety phenotypes and microglia activation. Individual FDP metabolites potently inhibited IL-1β production from microglia following stimulation with NLRP3-specific agonists, supporting anxiolytic properties of FDP observed in models of sleep deprivation involve inhibition of the NLRP3 inflammasome. The study further showed sleep deprivation alters the expression of the circadian gene *Bmal1*, which critically regulated NLRP3 expression and IL-1β production.

## Introduction

Several lines of research have suggested that improper immune function contributes to neuropsychiatric symptoms. Persistent immune responses have been identified in the serum of individuals with depression and anxiety disorders^1^. In addition, psychological stressors and chronic sleep deprivation increase plasma concentrations of inflammatory cytokines and are known risk factors for the development of both anxiety and major depressive disorder (MDD)^2,3^. Importantly, it has been noted that certain dietary regimens both reduce an individual’s susceptibility for neuropsychiatric disorders and reduce inflammatory markers^4^. Together, these findings suggest that dietary compounds can target immunological components of psychiatric disorders to promote resilience to psychological stress.

Recent studies have shown an association between heteromeric immune inflammasome complexes and behavioral phenotypes observed in mood disorders. Inflammasomes contain caspase-1 activity that facilitates proteolytic activation of pro-interleukin 1β and 18 (pro-IL-1β and −18) into their neuroactive forms which exert pleiotropic neurophysiological effects, and which can be neurotoxic^5^. In rodent models of stress-induced anxiety, activation of the NLR family pyrin domain-containing 3 (NLRP3) inflammasome and upregulation of IL-1β is noted in hippocampal and cortical brain regions and are critical for the onset of anxiety^6–9^. In patients with MDD and anxiety, NLRP3 gene expression is upregulated in peripheral monocytes^10,11^, while serotonergic anti-depressants downregulate inflammasome gene expression in MDD patients^10^. All inflammasomes contain domains that recognize specific bacterial and viral moieties and elicit immune responses by forming multimeric enzymatic complexes, but the NLRP3 inflammasome is also sensitive to oxidative stress^12^. This feature of the NLRP3 inflammasome is critical to consider given elevated levels of oxidative stress are prominent in brain regions governing emotion and memory following sleep deprivation^13^. In addition, two temporally distinct steps are critical in the assembly of the NLRP3 inflammasome: a transcriptional priming step, followed by activation-dependent on secondary cellular stress signals. The unique kinetic features of the NLRP3 inflammasome may therefore be one pathophysiological response that increases an individual’s susceptibility for neuropsychiatric symptoms following initial stressors^14^.

We previously identified a flavonoid-rich dietary preparation (FDP) composed of commercially available concord grape juice (CGJ), grape seed polyphenol extract (GSPE), and resveratrol (RSV), is effective in maintaining cognitive performance in models of psychological stress. Components of FDP attenuate depressive and anxiety phenotypes in a mouse model of repeated social defeat stress (RSDS) through reductions in the production of Interleukin 6 (IL-6) from peripheral leukocytes^15^. We demonstrated that FDP maintains cognitive performance in response to acute (single bout of 5 h) sleep deprivation through the promotion of protein translation in hippocampal neurons^16^. To further examine the molecular and cellular substrates of FDP responsible for its anxiolytic properties, we investigated the association between neuroimmune activity and FDP treatment in an established 6-day model of chronic sleep deprivation, which generates anxiety^17,18^.

In the present study, we show chronic sleep deprivation results in increased NLRP3 inflammasome expression, production of cytokines, and markers of microglia activation, accompanying an increase in anxiety phenotypes. Both anxiety and neuroinflammation were suppressed following the administration of FDP. Specific brain bioavailable metabolites of FDP inhibited NLRP3 inflammasome activation in vitro at physiologically active concentrations using cultured microglia. We further show that chronic sleep deprivation alters the expression of core circadian clock genes, while loss of the core circadian gene *Bmal1* caused loss of NLRP3 inflammasome regulation and IL-1β production.

## Results

### Anxiolytic and anti-inflammatory effects of FDP following chronic sleep deprivation

**Fig. 1:**
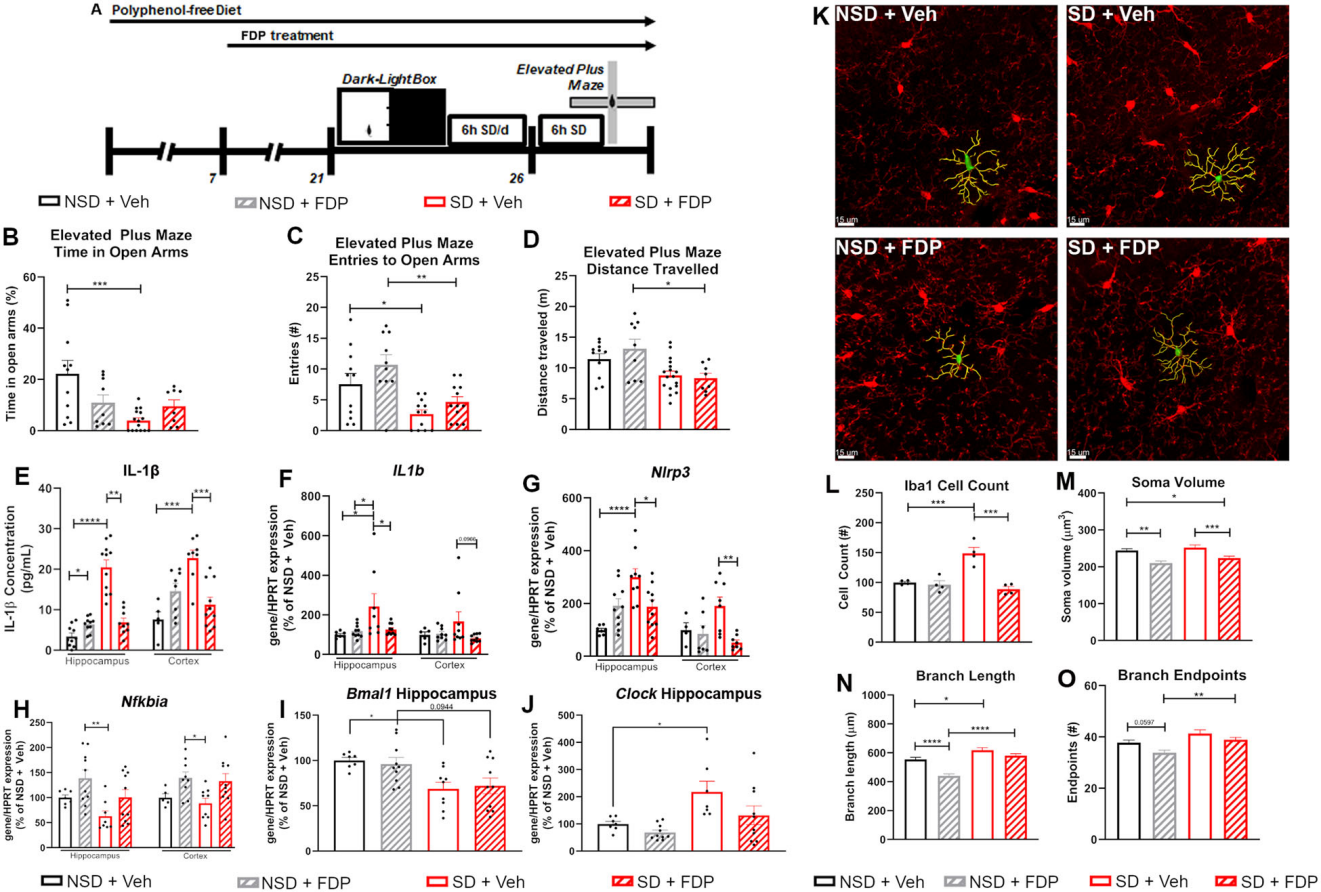
Treatment with a polyphenol-rich diet attenuates SD-associated anxiety and neuroinflammation. Analysis of behavior of wild-type mice treated with Vehicle or FDP, then tested in the Dark-Light Box task. Mice were then subjected to 6 hr SD for 6 days, then tested in the Elevated Plus Maze task. (**A**) Timeline of behavioral studies. (**B**) Percentage of Time spent in the Open Arms, (**C**) Entries into the Open Arms, and (**D**) Total Distance traveled during the Elevated Plus Maze task. *N* = 8–11 mice/group. (**E**) Production of IL-1β protein in the hippocampus and cortex. (**F**–**J**) mRNA expression of *IL1b* (**F**), *Nlrp3* (**G**), *Nfkbia* (**H**), *Bmal1* (**I**), and *Clock* (**J**) in the hippocampus and cortex. mRNA expression is compared to the expression of Hypoxanthine-guanine phosphoribosyltransferase (*Hprt*) and normalized to the mean of NSD + Veh mice. *N* = 7–10 mice/group for hippocampal samples, 5–10 for cortical samples. (**K**) Representative fluorescent images of CA1 hippocampal microglia. Red: Iba1. Green: Soma. Yellow: Branches. (**L-O**). Numbers (**L**), soma volume (**M**), total branch length per cell (**N**), and total branch endpoints per cell (**O**) of hippocampal microglia. *N* = 4 mice/group, >4 hippocampal sections studied per animal. For anatomical studies, >25 cells studied per section. *p < *0.05*, **p < *0.01*, ***p < 0.001, ****p < *0.0001* by Two-Way ANOVA with Tukey’s post-test.

To examine the effects of dietary flavonoids on chronic sleep deprivation-induced behavioral phenotypes, male wild-type C57BL/6J mice were randomly assigned to 2 groups and maintained on a diet lacking flavonoids for 1 week. Mice were then treated with regular drinking water (Vehicle, Veh) or FDP for an additional 2 weeks. Basal anxiety was then measured in a dark-light box consisting of a Dark Zone with opaque walls and an opaque ceiling and a Light Zone with opaque walls and exposure to bright lighting, at Zeitgeber Time 2 (ZT2; approximately 9:00 AM). Afterward, mice were subjected to either 6 h SD per day in an automated sleep deprivation system (Pinnacle Technology Inc.) or left undisturbed in their home cages (Non-SD, NSD) for six days. Immediately after the final bout of SD, the mice were assessed for SD-associated anxiety responses in an Elevated Plus Maze consisting of two arms that were exposed (Open Arms) and two arms with opaque walls (Fig. 1A). We did not detect a difference in the weight gain or diet and water consumption of Vehicle- or FDP-treated mice during pre-treatment. (Supplementary Fig. S1). In the dark-light box test, the amount of time spent in the light zone did not differ significantly between wild-type mice that were treated with Veh or FDP, although we did detect a decrease in the voluntary locomotion in the mice that were treated with FDP (Supplementary Fig. S2A-C). Mice subjected to six days of SD and treated with vehicle (SD + Veh) spent significantly less time in the open arms of the elevated plus maze than those that remained undisturbed (NSD + Veh) (Fig. 1B). Mice treated with FDP and subjected to SD (SD + FDP) did not differ in the time spent in the open arms of the elevated plus maze (Fig. 1B). SD + Veh mice also made significantly fewer entries into the Open Arms and NSD + Veh mice, which was attenuated in SD + FDP mice (Fig. 1C). SD + Veh mice also displayed a significant reduction in voluntary locomotion, but this decrease was not attenuated by FDP treatment (Fig. 1D).

We reported that treatment with dietary flavonoids attenuates neuroinflammation in a mouse model of stress-induced depression through regulation of IL-6 secretion^15^. We continued to explore the efficacy of FDP in attenuating stress-induced neuroinflammation in mice subjected to SD. We observed an increase of IL-1β secretion in the hippocampus and cortex of SD + Veh mice (Fig. 1E). Increased secretion of IL-1β in the hippocampus and cortex was attenuated by pre-treatment with FDP (Fig. 1E). We similarly detected an increase in the mRNA expression of *IL1b* and *Nlrp3* in the hippocampus of SD + Veh mice that was attenuated by pre-treatment with FDP (Fig. 1F, G). We also observed an SD-associated increase in the hippocampal expression of the DAMP *Hmgb1*, a TLR2/TLR4 ligand that upregulates *Nlrp3* (Supp Fig. 2D), although this upregulation was not significantly attenuated by FDP treatment. *Nlrp3* is a transcriptional target of NF-κB-mediated immune responses and its promoter is bound by p65 (RelA)^19^. NF-κB transcriptional activity is regulated in part by its interaction with nuclear factor of kappa light polypeptide gene enhancer in B cells inhibitor (IκB) proteins, which prevent the binding of NF-κB to DNA by masking its nuclear localization signal and preventing its translocation to the nucleus^20^. SD resulted in a decrease in the hippocampal expression of *Nfkbia* that was attenuated by pre-treatment with FDP (Fig. 1H). These SD-associated decreases in expression were not observed for *Nfkbib* or *Nfkbie*, although FDP treatment induced an increase of hippocampal and cortical *Nfkbib* (Fig. 1H, Sup Fig. 2E, F). These data suggest that SD induces inflammatory responses through increased NF-κB transcriptional activity and NLRP3 expression.

SD disrupts the activity of core components of the circadian clock, including the expression and activity of the transcription factors BMAL1 and CLOCK^21,22^. The circadian clock plays a critical role in the regulation of the innate immune response and its disruption exacerbates immune responses to stimulation with inflammatory agonists^23,24^. In particular, BMAL1 and CLOCK have been found to play antagonistic roles in the regulation of NF-κB-mediated transcriptional activity, with CLOCK promoting binding of p65 (RelA) to κB-binding sites and BMAL1 preventing binding through competitive interaction with CLOCK^25^. In the hippocampus and cortex of Veh + SD mice, we observed a decrease in the expression of *Bmal1* and a trending increase in the hippocampal expression of *Clock* (Fig. 1I, J). Pre-treatment significantly attenuated the upregulation of *Clock*, but not the downregulation of *Bmal1* (Fig. 1I, J). Altogether, these results suggest that SD may induce anxiety phenotypes through a mechanism involving disruption of the circadian clock and downstream NF-κB mediated transcriptional activity, in particular through dysregulation of *Bmal1* and *Clock*.

Given that chronic stress results in microglia proliferation, microglial ramification, and increased microglia soma size^26^, we hypothesized microglia activation would be evident in SD mice and anxiolytic properties of FDP involve its regulation of microglia phenotypes. In the hippocampus of SD + Veh mice, we detected increased numbers of Iba1-positive microglia, an event that was attenuated by pre-treatment with FDP (Fig. 1K, L). The CA1 hippocampal microglia of SD + Veh mice displayed greater ramification characterized by increased branch lengths and branch endpoints **(**Fig. 1N, O**)**. The activated morphology was attenuated by pre-treatment with FDP, which did not differ significantly from NSD + Veh mice. FDP pretreatment also resulted in smaller soma sizes compared to CSD + Veh mice (Fig. 1M). Microglia morphometric analysis supports that dietary flavonoids in FDP may provide resilience against CSD-induced anxiety by influencing microglia function.

### Select flavonoid metabolites inhibit NLRP3-associated inflammatory responses

**Fig. 2:**
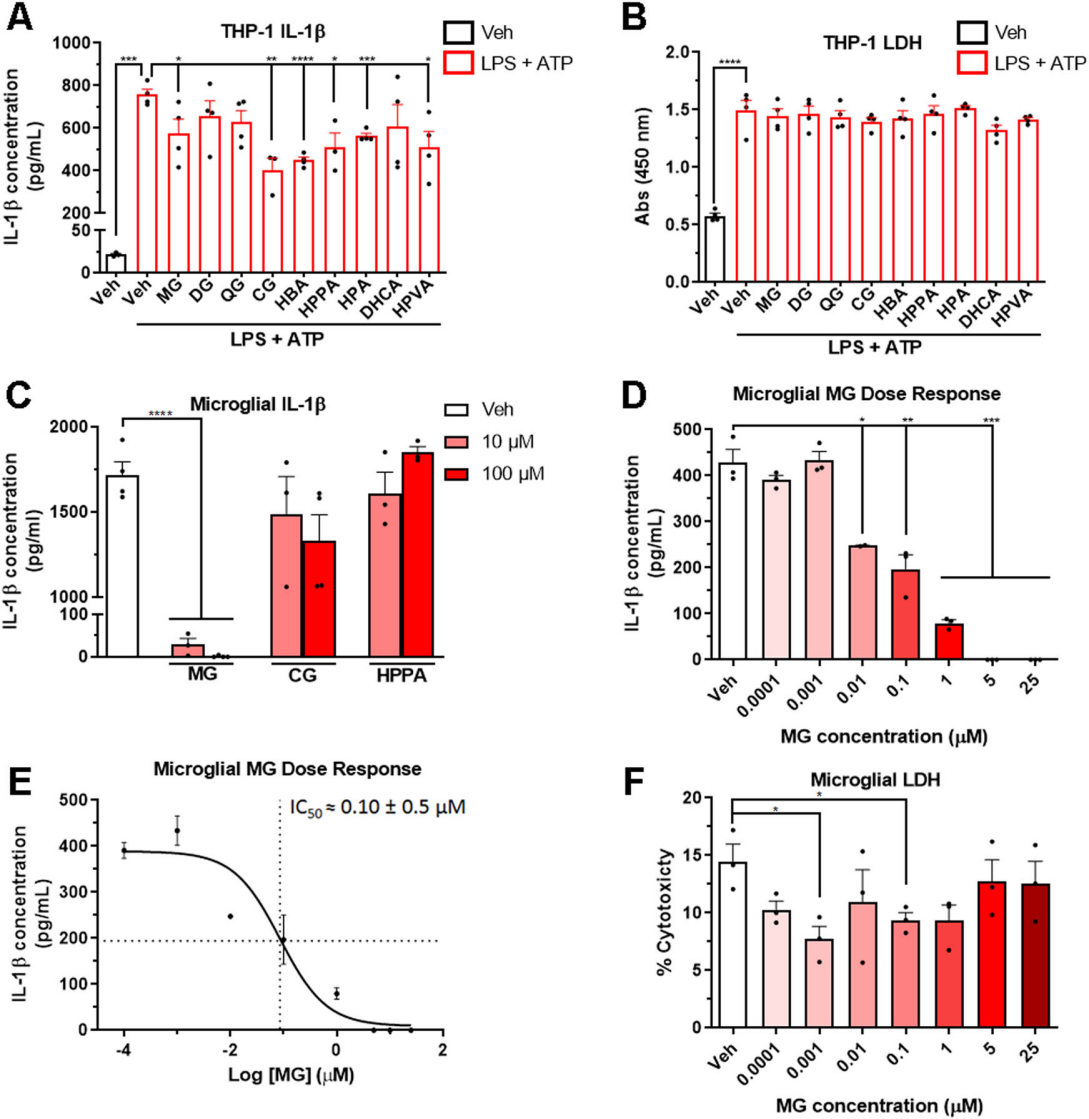
Select phenolic metabolites derived from FDP inhibit NLRP3-dependent production of IL-1β. (**A**) Secretion of IL-1β from THP-1 macrophages pre-treated with phenolic metabolites and then stimulated with LPS and ATP. MG: malvidin-glucoside. DG: delphinidin-glucoside. QG: quercetin-glucuronide. CG: Cyanidin-glucoside. HBA: 3-hydroxybenzoic acid. HPPA: 3-(3′-hydroxyphenyl)propionic acid. HPA: 3-hydroxyphenylacetic acid. DHCA: 3-(3′, 4′-dihydroxyphenyl)propionic acid. HPVA: 5-(4’-hydroxyphenyl)valeric acid. (**B**) Release of LDH from THP-1 macrophages pre-treated with phenolic metabolites then stimulated with LPS and ATP. (**C**) Secretion of IL-1β from mouse primary microglia pre-treated with phenolic metabolites and then stimulated with LPS and ATP. (**D**) Secretion of IL-1β in mouse primary microglia pre-treated with increasing concentrations of MG and stimulated with LPS and ATP. (**E**) Calculation of IC_50_ of IL-1β secretion in response to MG pretreatment and LPS and ATP stimulation. (**F**) Cytotoxicity of microglial cultures in response to MG pre-treatment, measured by LDH release. *N* = *3–4* biological replicates/group, performed in duplicate. *p < *0.05*, **p < *0.01*, ***p < 0.001, ****p < *0.0001* by Student’s *t-*test.

FDP contains a large variety of polyphenolic compounds. Previous high-throughput bioavailability studies have indicated that specific FDP-derived polyphenolic metabolites and phenolic acids accumulate in the brain after oral treatment with FDP (Supplementary Tables S1-S3)^27–29^. It is possible that specific metabolites may have an effect on NLRP3 activity whereas others may have no effect. Therefore, to minimize the possibility that the benefits of FDP treatment may be reduced by potential “cancellation” effects, we conducted in vitro screening studies of individual phenolic metabolites. To screen for metabolites that attenuate NLRP3-dependent inflammatory responses, we pre-treated THP-1 macrophages with brain bioavailable phenolic metabolites and measured secretion of IL-1β after stimulation with LPS and ATP. Of the nine brain-bioavailable metabolites screened, we found that malvidin-glucoside (MG), cyanidin-glucoside (CG), 3-hydroxybenzoic acid (HBA), 3-(3′-hydroxyphenyl)propionic acid (HPP), 3-hydroxyphenylacetic acid (HPA), and 5–4′hydroxyphenyl)valeric acid (HPVA) attenuated the production of IL-1β after stimulation with LPS and ATP (Fig. 2A). Treatment with metabolites did not increase the release of LDH, indicating a lack of toxicity in vitro (Fig. 2B). Follow-up screenings in mouse primary microglia determined that MG significantly inhibited LPS/ATP-induced production of IL-1β at a concentration of 10 µM (Fig. 2C), and a dose-response study in primary microglia estimated an IC_50_ of lL-1β secretion at 100 nM (Fig. 2D, E). No cytotoxicity in primary microglia was detected at concentrations of MG up to 25 µM (Fig. 2F). Follow-up studies in primary microglia from *Nlrp3*^*-/-*^ mice did not elicit increased secretion of IL-1β after stimulation with LPS + ATP, confirming that secretion of IL-1β in these cells is dependent on the activity of the NLRP3 inflammasome (data not shown). These data suggest that brain bioavailable flavonoid metabolites, and in particular MG, attenuate neuroinflammatory microglial responses through inhibition of NLRP3 inflammasome activity and secretion of the IL-1β.

### NLRP3 is a mediator of SD-associated anxiety phenotypes

**Fig. 3:**
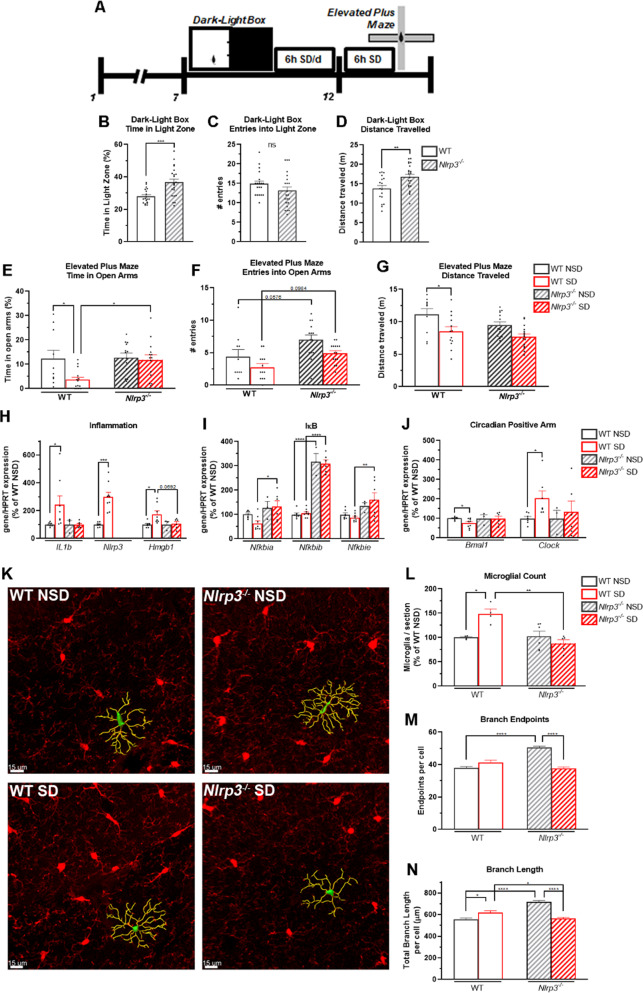
The NLRP3 inflammasome is a mediator of SD-associated anxiety and microgliosis. Analysis of behavior of wild-type and *Nlrp3*^*-/-*^ mice tested in the Dark-Light Box task. Mice were then subjected to 6 h SD for 6 days, then tested in the elevated plus maze task. (**A**) Timeline of behavioral studies. (**B**–**D**) Time spent in the Light Zone (**B**), Entries into the Light Zone (**C**), and Total Distance traveled in the dark-light box task (**D**). (**E**–**G**) Time spent in the open arms (**E**), Entries into the open arms (**F**), and total distance traveled in the elevated plus maze task (**G**). *N* = *19–22* mice/group for dark-light box, 10–14 mice/group for Elevated Plus Maze. (**H**) mRNA expression of the inflammation-related genes *IL1b, Nlrp3*, and *Hmgb1* in the hippocampus. (**I**) mRNA expression of the IκB genes *Nfkbia*, *Nfkbib*, and *Nfkbie* in the hippocampus. (**J**) mRNA expression of the circadian clock genes *Bmal1* and *Clock* in the hippocampus. *N* = *3–9* mice/group. (**K**) Representative hippocampal microglia. Red: Iba1. Green: Soma. Yellow: Branches. Scale bar: 15 μm. (**L**–**N**) Numbers (**L**), Total branch length per cell (**M**), and total branch endpoints of hippocampal microglia (**N**). *N* = 4–6 mice/group, >4 hippocampal sections studied per animal. For anatomical studies, >25 cells studied per section. *p < *0.05*, **p < *0.01*, ***p < 0.001, ****p < *0.0001* by Two-Way ANOVA with Tukey’s post-test.

The above data suggest that dietary flavonoids may attenuate SD-associated anxiety through inhibition of the NLRP3 inflammasome. To further investigate the role of the NLRP3 inflammasome in SD-associated anxiety, we tested wild-type (WT) C57BL/6J mice and mice lacking the *Nlrp3* gene (*Nlrp3*^*-/-*^) for basal anxiety in the Dark-Light Box task. Mice were then assigned to six days of SD or NSD at random, then tested for SD-associated anxiety responses in the Elevated Plus Maze task (Fig. 3A). In the Dark-Light Box task prior to SD, *Nlrp3*^*-/-*^ mice spent significantly more time in the Light Zone and exhibited an increase in voluntary locomotion compared to WT mice, suggesting reduced basal anxiety (Fig. 3B-D). WT mice subjected to SD (WT SD) spent significantly less time in the Open Arms of the Elevated Plus Maze and displayed a decrease in voluntary locomotion compared to wild-type mice subjected to NSD (WT + NSD; Fig. 3E-G). Interestingly, *Nlrp3*^*-/-*^ mice that were subjected to SD (*Nlrp3*^*-/-*^ SD) did not spend less time in the Open Arms or display SD-associated decreases in voluntary locomotion compared to *Nlrp3*^*-/-*^ mice not subjected to SD (*Nlrp3*^*-/-*^ NSD; Fig. 3E-G); *Nlrp3*^-/-^ SD mice spent significantly more time in the Open Arms than WT SD Mice. These data suggest that the NLRP3 inflammasome is a mediator of anxiety phenotypes induced by SD.

Thus, we examined the inflammatory responses of *Nlrp3*^*-/-*^ mice in the hippocampus and cortex. Unexpectedly, both *Nlrp3*^*-/-*^ NSD and *Nlrp3*^*-/-*^ SD mice displayed an increase of IL-1β production in the hippocampus compared to WT mice, suggesting the activation of compensatory mechanisms (Supp Fig. 3A). Despite the increased levels of IL-1β protein in the hippocampus, we did not observe an increase in the hippocampal expression of *IL1b* or *Hmgb1* in *Nlrp3*^*-/-*^ SD mice compared to WT mice (Fig. 3H). Expression of *Nlrp3* was not detected. SD also did not affect the expression of *Nfkbia*, *Nfkbib*, or *Nfkbie* in *Nlrp3*^*-/-*^ mice, and the expression of *Nfkbib* in *Nlrp3*^*-/-*^ mice was significantly increased compared to WT mice (Fig. 3I). Interestingly, we found that the expression of the core circadian clock genes *Bmal1*, *Clock*, *Cry1*, and *Nr1d1* was not disrupted in the brains of *Nlrp3*^*-/-*^ SD mice, compared to WT SD mice (Fig. 3J, Supp Fig. S3B).

We next determined the microglial response to SD in *Nlrp3*^*-/-*^ and WT mice. Compared to WT SD mice, *Nlrp3*^*-/-*^SD mice did not display microgliosis in the hippocampus (Fig. 3K, L). Unexpectedly, however, the microglia of *Nlrp3*^*-/-*^ NSD mice displayed a hyper-ramified morphology marked by increased branch endpoints and total branch length (Fig. 3M, N), as well as increased soma volume compared to WT NSD mice (Supp Fig. S3C). Each of these morphology changes was reversed in *Nlrp3*^*-/-*^ SD mice to resemble the microglia of WT SD mice (Fig. 3M, N, Supp Fig. S3C), suggesting that *Nlrp3* is a regulator of microglial activation states.

### Dietary flavonoids inhibit microgliosis associated with disruption of the circadian clock

**Fig. 4:**
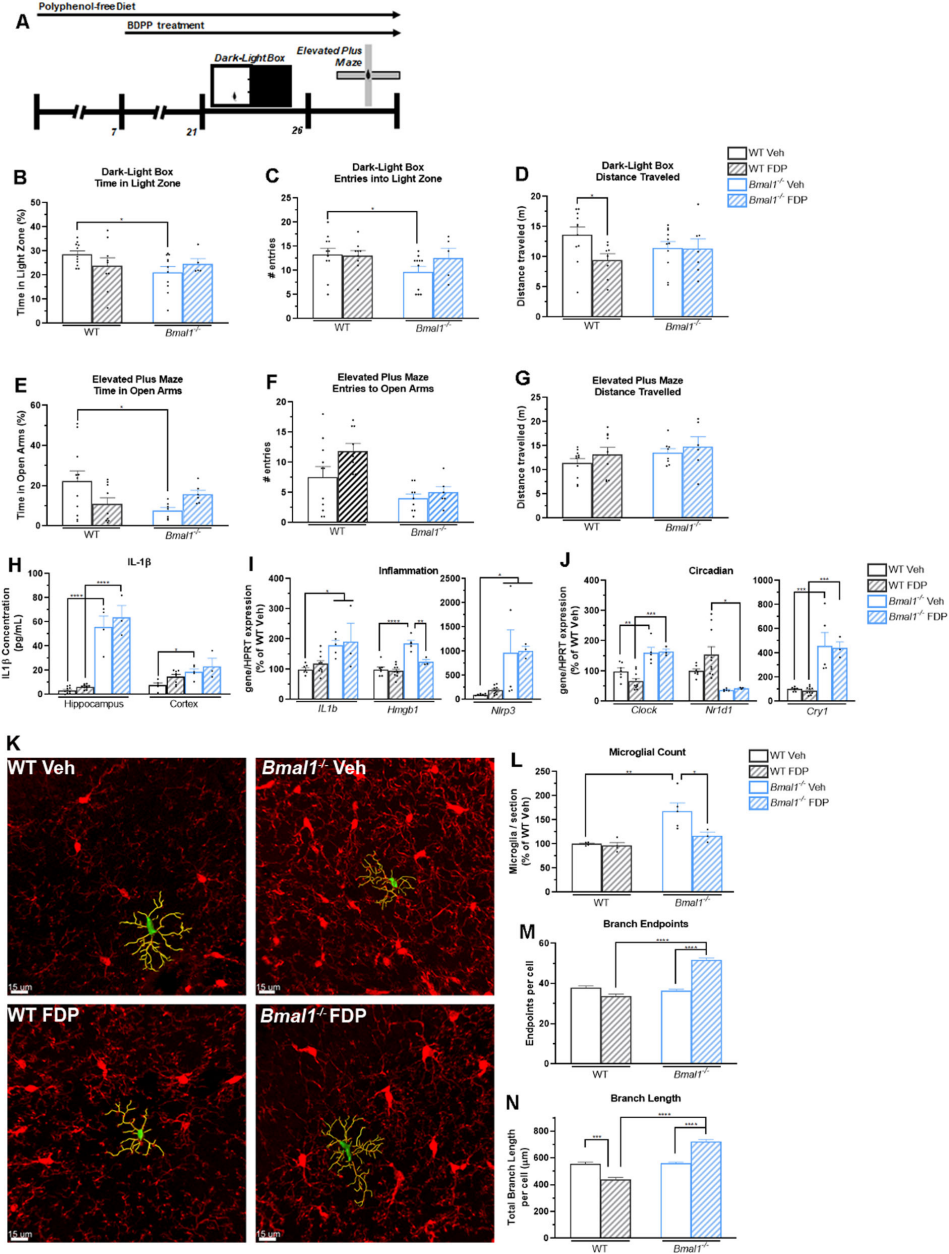
Treatment with polyphenols attenuates anxiety but not IL-1β production induced by disruption of the circadian clock. Analysis of behavior of wild-type and *Bmal1*^*-/-*^ mice tested in the dark-light box and elevated plus maze task. (**A**) Timeline of behavioral studies. (**B-D**) Time spent in the light zone (**B**), Entries into the light zone (**C**), and total distance traveled in the dark-light box task (**D**). (**E**–**G**) Time spent in the open arms (**E**), Entries into the open arms (**F**), and total distance traveled in the elevated plus maze task (**G**). *N* = 5–11 mice/group. (**H**) IL-1β production in the hippocampus and cortex. *N* = 3–10 mice per group. (**I**) mRNA expression of the inflammation-related genes *IL1b, Nlrp3*, and *Hmgb1* in the hippocampus. (**J**) mRNA expression of the circadian clock genes *Clock, Cry1*, and *Nr1d1* in the hippocampus. *N* = 3–10 mice/group. (**K**) Representative fluorescent images of CA1 hippocampal microglia. Red: Iba1. Green: Soma. Yellow: Branches. Scale bar: 15 μm. (**L**–**N**) Numbers (**L**), total branch length per cell (**M**), and total branch endpoints per cell (**N**) of hippocampal microglia. *N* = 3–5 mice/group, >4 hippocampal sections studied per animal. For anatomical studies, >25 cells studied per section. *p < *0.05*, **p < *0.01*, ***p < 0.001, ****p < *0.0001* by Two-Way ANOVA with Tukey’s post-test.

Mood disorders such as anxiety are linked to disruptions in the molecular circadian clock. Knockdown of *Bmal1* expression alone induces anxiety phenotypes and disruptions in the rhythmic expression of core circadian clock proteins^30,31^. Our above data suggest that disruption of the circadian clock induced by SD upregulates the expression of *Nlrp3*. To examine the effect of flavonoid treatment on a model of circadian disruption, we placed both WT mice and mice in which the *Bmal1* gene is deleted (*Bmal1*^*-/-*^) on a diet lacking in flavonoids and treated then with FDP or Veh. Mice were then tested for basal anxiety in a dark-light box task, then were tested in the elevated plus maze task in the absence of SD (Fig. 4A). We did not detect a difference in the weight gain or diet and water consumption of Veh- or FDP-treated mice during pre-treatment. (Supplementary Fig. S4). In the Dark-Light Box task, *Bmal1*^*-/-*^ mice that were treated with Veh (*Bmal1*^*-/-*^ Veh) spent significantly less time in the Light Zone compared to WT mice treated with Veh (WT Veh; Fig. 4B, C). These decreases were attenuated by treatment with FDP. Unlike WT mice, *Bmal1*^*-/-*^ + FDP mice did not display a reduction in voluntary locomotion (Fig. 4D). *Bmal1*^*-/-*^ Veh mice spent significantly less time in the open arms of the elevated plus maze compared to WT Veh mice (Fig. 4E), although we did not detect a significant reduction in the number of entries into the open arms or a reduction in voluntary locomotion (Fig. 4F, G). *Bmal1*^*-/-*^ mice treated with FDP (*Bmal1*^*-/-*^ FDP) did not display a decrease in the amount of time spent in the open arms (Fig. 4E).

As deletion of *Bmal1* resulted in anxiety phenotypes, we examined the production of IL-1β and expression of inflammatory genes in the hippocampus of both WT and *Bmal1*^*-/-*^ mice. In both the hippocampus and cortex *Bmal1*^*-/-*^ Veh mice, we observed an increase in the production of IL-1β that was not attenuated by treatment with FDP (Fig. 4H). We similarly detected an increase in the expression of *IL1b, Nlrp3*, and *Hmgb1* in the hippocampus of *Bmal1*^*-/-*^ Veh mice (Fig. 4I). Although treatment with FDP was able to attenuate the expression of *Hmgb1*, no significant effect was detected on the expression of *IL1b* or *Nlrp3* (Fig. 4I). Interestingly, we did not detect a significant change in the expression of *Nfkbia* or *Nfkbib* in the hippocampus of *Bmal1*^*-/-*^ Veh or *Bmal1*^*-/-*^ FDP mice, although the expression of *Nfkbie* was increased in the hippocampus of both *Bmal1*^*-/-*^ Veh and *Bmal1*^*-/-*^ FDP mice (Supp Fig. S5A). Similar to WT SD mice in Fig. 1, we observed a significant disruption in other core circadian clock genes in the brains of both *Bmal1*^*-/-*^ Veh and *Bmal1*^*-/-*^ FDP mice. In the hippocampus of *Bmal1*^*-/-*^ mice, we observed an increase in the expression of both *Clock* and *Cry1*, and a decrease in the expression of *Nr1d1* (Fig. 4J).

We next examined the microglial response to dietary FDP in the brains of WT and *Bmal1*^*-/-*^ mice. In the hippocampus of *Bmal1*^*-/-*^ Veh mice, we observed an increase in Iba1-positive microglia that was attenuated by FDP treatment (Fig. 4K, L). Unexpectedly, the ramification state of *Bmal1*^*-/-*^ Veh mice was similar to that of WT Veh mice, without any significant changes in Branch Endpoints, Total Branch Length, or Soma Volume (Fig. 4M, N, Supp Fig. 5B). Treatment with FDP induced an increase in the Branch Endpoints, Total Branch Length, or Soma Volume in the microglia of *Bmal1*^*-/-*^ FDP mice (Fig. 4M, N, Supp Fig. 5B). Altogether, these results indicate that disruption of the circadian clock through the deletion of the core circadian clock gene *Bmal1* induces anxious and inflammatory phenotypes that are only partially attenuated by dietary flavonoids.

## Discussion

These results expand upon our previous findings that FDP treatment preserves cognitive function in both models of stress^15,32^ and acute sleep deprivation^16^. In the present study, we further emphasize the beneficial effects of FDP treatment in models of stress, finding that administration of FDP to mice subjected to chronic sleep deprivation reduced anxiety by suppressing microglia activation and NLRP3 inflammasome activity. As individual metabolites derived from FDP inhibited IL-1β production and NLRP3-deficit mice show suppressed anxiety in response to chronic sleep deprivation, this supports the conclusion that anxiolytic effects of FDP were in part due to suppressed NLRP3 activity. An additional finding here is that chronic sleep deprivation alters the hippocampal expression of the circadian gene *Bmal1*, which has a critical function in suppressing *Nlrp3* gene expression.

### Anxiolytic Properties of FDP Involve Inhibition of Interleukin 1β and NLRP3 Inflammasome Activity

The results of these chronic sleep deprivation studies show a critical role of NLRP3 inflammasome signaling and IL-1β production in mouse hippocampus and cortex for the generation of anxiety. This work is consistent with studies that show NLRP3 inflammasome activation in hippocampal and frontal cortical regions of mice also contributes to anxiety in models of chronic social stress^6^ and that brain IL-1β recruits splenic monocytes into the mouse brain in social stress models, leading to the onset of anxiety^7,8^. Chronic sleep deprivation and repeated social stress exert distinct physiological effects and impact different neurobiological circuits. Yet, elevated cortisol, inflammation, and anxiety occur in both model systems^33,34^. Our studies support the principle that increased sensitivity to IL-1β production is an additional shared pathophysiological pathway in both stress model systems, which is in part responsible for their anxiety phenotypes. The cellular and molecular substrates IL-1β interacts with to generate anxiety needs to be validated. Yet, IL-1β clearly plays a role in the molecular pathophysiology of stress-induced anxiety, because interleukin-1 receptor 1 (IL-1R) deficient mice are resistant to anxiety phenotypes^35^. The interdependence between IL-1R and anxiety may involve the observed phenomenon in which IL-1β promotes expression of IL-1 receptor type 1 and the accessory coreceptor AcP, rather than the synapse promoting AcPb subunit in hippocampal neuronal cultures, leading to impaired long-term potentiation^36^. Thus, the ability of FDP to reduce brain IL-1β in response to chronic sleep deprivation and the role of IL-1R in generating anxiety support anxiolytic properties of FDP.

### Individual Metabolites of FDP Inhibit Interleukin-1β Production from Microglia

This work extends previous studies that identified specific metabolites of FDP that exhibited immuno-suppressive properties in a model of social stress^15^. In previous studies, FDP metabolites dihydrocaffeic acid (DHCA) and malvidin-glucoside (MG) prevented depressive phenotypes in response to social stress by respectively inhibiting IL-6 monocyte production and preventing maladaptive synapse formation in the nucleus accumbens (NAc). Both MG and DHCA are present at nanomolar concentrations in the brain following dietary administration to mice^37^. Here, the findings that MG inhibits IL-1β at sub-micromolar concentrations from murine microglia in response to LPS provides evidence that FDP metabolite concentrations in the brain following oral administration are sufficient to exert physiological effects against brain IL-1β production following sleep deprivation. Although the FDP metabolites cyanidin-glucoside (CG) and HPPA inhibited IL-1β production from THP-1 human monocytes more effectively than MG, MG was more effective at inhibiting IL-1β from murine microglia following LPS + ATP stimulation. This discrepancy may be related to the differential mechanism of NLRP3 inflammasome regulation observed in different immune cells^38^. Additionally, the metabolites queried may inhibit other inflammasomes, as multiple inflammasome complexes result in caspase-1 mediated activation of IL-1β^39^. For example, NLRP3 and NLRC4 are recruited to the same inflammasome complex to elicit inflammatory responses to bacterial infection or flagellin stimulation^40,41^. The observation that *Nlrp3*^-/-^ mice do not exhibit anxiety phenotypes following sleep deprivation support that FDP metabolites reduced IL-1β in sleep deprivation models by inhibiting the NLRP3 inflammasome. The NLRP3 inflammasome is also functionally distinct from other inflammasomes through its sensitivity and activation in response to endogenous sterile stress signals generated during cellular stress^19,42,43^. As our study and others have described, chronic sleep deprivation generates several sterile stress signals that are able to activate the NLRP3 inflammasome including mitochondrial oxidative stress and HMGB1^44^. The functional capacities of the NLRP3 inflammasome support its role as the primary source of IL-1β immune responses generated in response to sleep deprivation stress signals. As MG exhibits both a strong safety profile and a potent ability to inhibit IL-1β production, it should be further validated as a monoagent therapeutic in disorders in which IL-1β exerts a pathological effect.

While our studies did not directly examine markers of mitochondrial oxidative stress, treatment with GSPE (a component of FDP) preserves memory function in a mouse model of vascular dementia through alleviation of oxidative stress^45^. Individual metabolites that potently inhibited the production of IL-1β in vitro, including MG and CG, upregulate superoxide dismutases and phase II enzymes such as Glutathione S-transferases, while inhibiting the expression of xanthine oxidase^46,47^. Metabolites derived from FDP may have pleiotropic effects in the preservation of cognitive function during stress including the inhibition of inflammasome activity as well as amelioration of oxidative stress, thus inhibiting the activation of microglia^48^.

The plasma and brain bioavailability of phenolic metabolites derived from FDP is regulated by the gut microbiota. Dietary polyphenols commonly occur as oligomers and conjugates to sugar and organic acids that are poorly absorbed in the small intestine^49^. Colonic bacteria de-conjugate and further modify polyphenols to produce bioactive phenolic metabolites such as anthocyanidins (MG and CG) and phenolic acids (HBA, HPPA) through a number of catabolic mechanisms including deglycosylations, C-ring cleavage, and dehydroxylation^50^. Confirming the necessary role of the gut microbiota in the promotion of cognitive function by FDP, co-treatment of mice with a combination of antibiotics significantly reduced the bioavailability of FDP-derived metabolites and abolished the pro-resilience effects of FDP in a paradigm of sleep deprivation^51^. Studies in humanized gnotobiotic mice further demonstrate that the composition of the gut microbiota influences the bioavailability of phenolic acids after treatment with polyphenols^52^. Collectively, these findings support studies to identify bacterial strains that increase the bioavailability of bioactive compounds and the formulation of synbiotics, optimized combinations of probiotic bacterial strains and dietary polyphenols, as a potential therapeutic paradigm for the treatment of psychological disorders^49^.

The strong safety and efficacy profiles of FDP and derived metabolites support further clinical trials in patients for the preservation of cognitive function in mild cognitive decline and stress-related inflammation^15,48^. Examination of the benefits of CGJ in a Phase I/IIA trial in patients with Gulf War Illness found that treatment was well tolerated and resulted in improvements in executive functioning^53^. Treatment with grape-derived polyphenols from a variety of sources, including CGJ, in patients suffering from mild age-related cognitive decline, resulted in benefits in working memory and improved verbal learning^54,55^. The anti-inflammatory and anti-oxidant properties of FDP-derived metabolites, demonstrated in vitro, in vivo using models of stress^15,16,46,47^, have been recapitulated in preclinical and clinical trials^53–55^. Our current study, along with these preclinical successes demonstrate that FDP may serve as a potential therapeutic for addressing the immunological pathologies associated with mood disorders and cognitive decline.

### FDP Influences Microglia Morphology and Activity

The suppression of an inflammatory microglia phenotype by individual FDP metabolites using an in vitro system was complemented by morphometric analysis of microglia in mice that underwent chronic sleep deprivation. Microglia are the resident innate immune cells in the brain and regulate synaptic steady-states via innate immune signaling^56,57^. Thus, whether microglia exist in either a ramified surveillant phenotype—one associated with synaptic phagocytosis—or an ameboid phenotype with short, thicker processes, a larger soma, and immune activity has implications for neuronal cytoarchitecture and behavior^58^. Indeed, an activated microglia phenotype is associated with anxiety and depression in mouse models of social stress^59^. Our results confirmed that chronic sleep deprivation results in morphological evidence of microglia activation and enhanced microglial phagocytosis of synaptic elements^18^. In addition to maintaining a surveillant microglia phenotype in the hippocampus in response to chronic sleep deprivation, FDP treatment also reduced markers of microglia activation in non-sleep deprived mice, suggesting FDP may help maintain noninflammatory microglia phenotypes under non-stressed conditions. These studies also demonstrated that as observed in models of chronic stress, hippocampal microglia also undergo proliferation in response to chronic sleep deprivation, as assessed by increased IBA1 + microglia^60^. How increased microglia densities affect dendritic morphology, neuronal activity, and behavior in response to sleep deprivation is unknown. Our study did not examine the effect of chronic sleep deprivation on synaptic structures in the hippocampus and frontal cortex; it is unknown if FDP suppresses anxiety through synaptic mechanisms. Such studies are critical, as 72 h sleep deprivation caused defects in synaptic pruning by reducing microglia phagocytosis^61^ and FDP treatment prevented increased PSD95 synapse production in the NAc of mice exposed to chronic social stress^15^.

Microglia phenotype and activity are influenced by extracellular immune molecules^62,63^. Mice with transgenic overexpression of brain IL-1 receptor antagonist do not undergo hippocampal microglia proliferation, nor exhibit increased soma volume, and branch length immediately following chronic unpredictable stress^64^. Our results support a similar association between IL-1β production and microglia activation and proliferation in a model of chronic sleep deprivation. Not only did FDP treatment simultaneously suppress IL-1β production in the brain and promote anti-inflammatory microglia phenotypes, but *Nlrp3*^*-/-*^ mice exposed to sleep deprivation did not exhibit evidence of microglia activation or proliferation. A requirement for NLRP3 to generate active microglia has been reported in models of Alzheimer’s disease^65^. In addition, this study, to our knowledge, is the first to examine microglia morphology in *Nlrp3*^*-/-*^ mice, finding significantly more complex microglia processes in wild-type mice unexposed to stimulus. While the canonical function of NLRP3 involves its role in forming a multimeric inflammasome protein complex, studies show that NLRP3, independently, acts a transcription factor that regulates immune cell phenotypes^66^. Heightened microglia ramification in *Nlrp3*^*-/-*^ mice may be related to depressed IL-1β signaling, but it also may involve an undefined role for NLRP3 as a transcription factor in microglia. The influence NLRP3 has on microglia phenotypes through transcriptional pathways requires further study. Together, our microglia analysis suggests inflammasome production of IL-1β promotes microglia phenotype responses in models of sleep deprivation and serves as a molecular substrate that can be targeted with FDP metabolites to promote resilience to anxiety.

### BMAL1 Impairment Leads to Altered NLRP3 Inflammasome and IL-1β Signaling

These studies extended into the potential upstream cause of dysregulated NLRP3 inflammasome activity following chronic sleep deprivation. Sleep deprivation altered the expression of the core circadian genes, *Bmal1* and *Clock*, while concurrently increasing the expression of *Nlrp3* and production of IL-1β. The simultaneous dysregulation of *Bmal1* and *Nlrp3* expression supports a previously undefined relationship between *Bmal1* and *Nlrp3*. Numerous investigations have found circadian proteins regulate the expression of immune genes^67^. Of note, the inflammatory genes *Tlr8* and *Ccl2* exhibit antiphase and phase-matched oscillations relative to the cellular expression of *Bmal1* and *Clock*^68^. It is possible BMAL1 regulates *Nlrp3* expression by interacting with NF-κB, which has putative binding sites on the *Nlrp3* promoter^69^.

Further, BMAL1 drives the expression of RORα, a positive regulator of the NF-κB inhibitory protein IκBα^24^. Regulation of *Nlrp3* by *Bmal1* via NF-κB mechanisms is consistent with our findings that chronic sleep deprivation results in downregulation of mRNA expression of IκBα. These findings add to the literature suggesting that alterations to the molecular architecture or frequency of oscillating circadian genes may be a vector for altered behavioral phenotypes through immune function^70,71^. In addition, long-term consequences, such as accelerating aging^72^ and susceptibility to autoimmune disorders^73^ are observed with circadian dysregulation. Future studies should investigate the relationship, if any, between polyphenol rich diets associated with extended life spans^74^ and the maintenance of proper immune function by circadian proteins.

Sleep deprivation disrupts a multitude of physiological processes—one of which is properly regulated immune function. Our study provides the incentive to further research and develop targeted dietary approaches that provide resilience against the neurobiological consequences of impaired immune function, and its psychiatric effects.

## Materials and Methods

### Materials

Grape Seed Polyphenol Extract (GSPE; MegaNatural®-BP Polyphenolics, Warehouse, UPC: 603573579173), Concord Grape Juice (CGJ; Welch Foods, Concord MA), and Resveratrol (RSV; ChromaDex, Irvine CA) were obtained commercially. Malvidin-glucoside, delphinidin-glucoside, quercetin-glucuronide, and cyanidin-glucoside (Extrasynthesis, Genay Cedex, France); 3-hydroxybenzoic acid, 3-(3′-hydroxyphenyl)propionic acid 3-hydroxyphenylacetic acid, 3-(3′, 4′-dihydroxyphenyl)propionic acid, and 5-(4′-hydroxyphenyl)valeric acid (Sigma-Aldrich, St. Louis MO) were obtained commercially and were analyzed by LC-MS and archived as previously reported in compliance with NCCIH Product Integrity guidelines^75,76^. Unless specified otherwise, cell culture reagents were obtained from ThermoFisher Scientific (Waltham, MA) and antibodies were obtained from Abcam (Cambridge, MA).

### Subjects

*Bmal1*^*-/-*^ and *Nlrp3*^*-/-*^ mice were obtained from the Jackson Laboratory (#009100 and #021302, respectively). Mice were given food and water ad libitum and were socially housed on a 12:12-h light/dark cycle with lights on at 07:00 h in a temperature-controlled (20 ± 2 °C) vivarium. Male mice were 8–12 weeks old at the time of behavior. All procedures were approved by the Institutional Animal Care and Use Committee of the Icahn School of Medicine at Mount Sinai.

### FDP treatment

Mice were randomly grouped into two groups: one group receiving regular drinking water (vehicle), and the other treated with FDP composed of GSPE, RSV, and CGJ, delivered through drinking water for 2 weeks prior to experiments. The daily intake of GSPE was 200 mg kg^−1^ body weight (BW), RSV was 300 mg kg^−1^ BW, and CGJ was 1 mL d^−1^.

### Behavior assays

All behavioral assays were conducted 2 h after the beginning of the light cycle of the day (Zeitgeber Time 2; ZT2). The dark-light box test was conducted in a room with 500 lux bright lighting. The dark-light box consisted of two 40 cm × 40 cm × 40 cm Plexiglas boxes with a hole cut between them to allow for passage between the boxes. The light zone had opaque white walls and an open ceiling, and the dark zone had black opaque walls and a black opaque ceiling. Both boxes were filled with bedding material. Animals were placed within the apparatus facing away from the Dark Box and were allowed to freely explore the apparatus for 10 min and then were returned to home cages. The elevated plus maze test was conducted in a separate room with 300 lux lighting. The elevated plus maze (Noldus, Wageningen, The Netherlands) consisted of a four-armed apparatus with 40 cm-long gray arms. Two arms were exposed (Open Arms) and two were flanked by opaque gray plastic walls (Closed Arms). The animal was placed within the center of the apparatus facing towards an open arm and was allowed to freely explore the apparatus for 5 min and then was returned to its home cage. All test trials were video recorded, tracked, and analyzed with ANY-Maze tracking software (v 6.0; Stoelting, Wood Dale, IL). Mice were habituated to the testing room for 30 min prior to the beginning of the trials. All tests were conducted by the same experimenter.

### Sleep deprivation

Mice were subjected to 6 h of sleep deprivation, per day, for 6 days, immediately following the initial behavioral assay in the dark light box apparatus. Mice were placed in a stand-alone sleep deprivation system (Pinnacle Technology Inc.) and provided food and either drinking water or FDP.

### Assessment of gene expression in total RNA from mouse tissue

RNA from mouse hippocampal and cortical tissue was isolated using RNeasy Mini Kit (QIAGEN, Hilden, Germany) according to the manufacturer’s instructions. In total 500 ng of RNA was reverse transcribed using High-Capacity cDNA Reverse Transcription Kit (ThermoFisher) according to the manufacturer’s instructions. qRT-PCR was performed to measure the expression of genes of interest. Gene expression was measured in 4 replicates through PowerUp SYBR Green Master Mix (ThermoFisher) using an ABI PRISM 7900HT Sequence Detection System. Hypoxanthine phosphoribosyltransferase (*Hprt*) expression level was used as an internal control and data was normalized using the 2 − ΔΔ*Ct* method^77^. Levels of target gene mRNA was expressed relative to those of NSD + Veh mice for in vivo studies and to cells treated with Vehicle for in vitro studies. Primers used in this study were designed using Primer-BLAST software^78^ and are listed in Supplementary Table S4. qPCR and data analysis was performed at the qPCR CoRE at the Icahn School of Medicine at Mount Sinai.

### Immunofluorescence

After 1.5 h of completion of behavioral trials, mice were anesthetized with 100 mg kg^−1^ ketamine and 10 mg kg^−1^ xylazine and perfused transcardially with cold PBS followed by 4% paraformaldehyde in PBS. Brains were removed and kept in 4% PFA at 4 °C overnight prior to washing with cold PBS. Coronal slices (40 µm thick) were taken using a vibratome (Leica, Wetzlar, Germany) and collected in cold PBS + 0.02% sodium azide. For immunostaining, each slice was washed three times with cold PBST (PBS + 0.1% Triton X-100) for 10 min, then placed in PBST with 5% normal goat serum for 1 h. Slices were then incubated with primary antibody diluted in PBST with 5% goat serum at 4 °C for 24 h (Rabbit anti-Iba1 1:500, Abcam ab178846). The next day, slices were washed three times for 10 min in cold PBST, followed by 2 h incubation in secondary antibody diluted in PBST with 5% goat serum (Goat anti-Rabbit AlexaFluor568 1:250, Abcam ab175471). Slices were washed three times for 10 min in cold PBST, followed by mounting and coverslipping on microscope slides using VectaShield containing DAPI (H-1500, Vector Laboratories, Burlingame CA). Images of coronal sections were taken on a Zeiss LSM880 Airyscan confocal microscope (Oberkochen, Germany) using an X20/0.8 NA air immersion objective controlled by Zeiss Zen Black software. For 3D analysis, z-stack images were obtained by capturing an image every 0.7 μm covering the entire 40 μm-thick section. Images were deconvoluted using AutoQuant X3.1 (Media Cybernetics, Rockville MD) and 3D analysis was performed using Imaris 9.1.2 (Bit Plane Inc, Concord MA) using the surface tool to reconstruct the soma and the filaments tool to reconstruct the branches. Counts of microglia were analyzed using FIJI software^79^. Microscopy and image analysis was performed at the Microscopy CoRE at the Icahn School of Medicine at Mount Sinai.

### THP-1 macrophage cultures

THP-1 human monocytic cultures were obtained from ATCC (TIB-202, Manassas, VA) and were cultured by the manufacturer’s recommendations. To differentiate into macrophages, THP-1 cultures were seeded on 24-well plates and treated with 100 ng/mL Phorbol 12-myristate 13-acetate (Sigma-Aldrich) for 48 h. Cells were then washed twice with RPMI-1640 medium, then cultured in RPMI-1640 + 10% FBS + 1% Penicillin-Streptomycin for 24 h. Cells were then treated with phenolic metabolites for 24 hr, then 1 µg/mL LPS (O55:B5) for 5 h, followed by 5 mM ATP for 40 min. For initial screenings of efficacy, THP-1 cells were treated with phenolic acids (HBA, HPPA, HPA, DHCA, HPVA) at a concentration of 10 μM and polyphenolic compounds (MG, DG, QG, CG) at a concentration of 500 nM. Cellular supernatant was then harvested for analysis of secreted cytokines. Viability of cell cultures was measured using CytoTox 96 NonRadioactive Cytotoxicity Assay (Promega, Madison WI).

### Mouse primary microglial cultures

Cortices from 1 to 3 day-old C57BL/6J mouse pups were isolated, digested, and seeded at a density of 8 cortices per 10 mL culture dish. Every three days, medium (DMEM + 10% FBS + 1% penicillin-streptomycin) was replenished. After 3 weeks, mixed glial cultures reached confluence and were isolated by mild trypsinization as previously described^80,81^. Briefly, cells were washed with culture medium without FBS and treated with a mixture of trypsin (0.25% without EDTA) and DMEM-F12 medium in a 1:3 ratio. After 40 min incubation, mixed glial cells detached and left a layer of microglia attached to the bottom of the culture dish. Pure microglia were isolated by 15 min incubation with trypsin (0.05% with EDTA) at 37 °C followed by gentle shaking. Cells were counted and seeded in 24-well plates at a density of 7.5 × 10^4^ cells/wells. Cells were treated with phenolic metabolites for 24 h, then 500 ng/mL LPS (O55:B5, Sigma-Aldrich) for 3 h and 5 mM ATP (Sigma-Aldrich) for 30 min. Cellular supernatant was then harvested for analysis of secreted cytokines. Viability of cell cultures was measured using CytoTox 96 NonRadioactive Cytotoxicity Assay.

### Analysis of cytokines

For investigation of the effect of FDP on the expression of cytokines, a subset of mice were sacrificed and brain regions and plasma were harvested. Brain regions were lysed with 1X Cell Lysis Buffer supplemented with 1 mM PMSF (Cell Signaling, Danvers MA) and Protease Inhibitor Cocktail (Sigma-Aldrich, cat no. 11873580001). Brain tissue IL-1β was measured with Mouse IL-1 beta/IL-1F2 DuoSet ELISA Kit (R&D Systems, Minneapolis MN) according to the manufacturer’s instructions. For mouse primary microglia and human THP-1 macrophages, tissue culture supernatant was harvested for analysis of secreted cytokines. In human THP-1 cells, IL-1β was measured with Human IL-1 beta/IL-1F2 DuoSet ELISA Kit (R&D Systems) according to the manufacturer’s instructions.

### Statistical analysis

All values are presented as mean and standard error of the mean (s.e.m.). For all studies, Two-Way ANOVA followed by Tukey’s post-test (confidence interval = 95%) or unpaired two-tailed Student’s *t-*test with Welch’s correction was used (confidence interval = 95%). In all studies, outliers (>2 SD from the mean) were excluded. All statistical analysis was performed using GraphPad Prism 8 software (GraphPad Software, San Diego CA). *p < *0.05*, **p < *0.01*, ***p < 0.001, ****p < *0.0001*, ns not significant.

## Supplementary information

Supplemental Table and Figure Legends

Supplementary Figure S1

Supplementary Figure S2

Supplementary Figure S3

Supplementary Figure S4

Supplementary Figure S5

Supplementary Table S1

Supplementary Table S2

Supplementary Table S3

Supplementary Table S4

## Data Availability

The data that support the findings of this study are available from the corresponding author upon reasonable request.
