# Supplementary material for: Anxiolytic effects of NLRP3 inflammasome inhibition in a model of chronic sleep deprivation
Source: Transl Psychiatry. 2021 Jan 14;11:52. doi: 10.1038/s41398-020-01189-3 (PMC7809257; doi:10.1038/s41398-020-01189-3)
Supplement: Supplementary file 1 — Supplemental Table and Figure Legends [file 41398_2020_1189_MOESM1_ESM.docx]

**Supplementary Figure S1: Physiological monitoring data. (A)** Body weight (g), diet consumption (g/day) and liquid consumption of FDP or vehicle (mL/day) in wild-type non-sleep deprived vehicle-treated mice (WT NSD + Veh), non-sleep deprived FDP-treated mice (WT NSD + FDP), sleep-deprived vehicle-treated mice (WT SD + Veh) and sleep-deprived FDP-treated mice (WT SD + FDP) that were then tested for behavior in the Dark-Light Box and Elevated Plus Maze tasks. **(B-E)** Graphic presentation of **(B)** body weight (g), (**C**) body weight change/week (g), (**D**) diet consumption (g/day) and (**E**) liquid consumption (mL/day) across the 3-weeks treatment regimen. Data is mean ± SEM (Repeated measures ANOVA with Tukey’s post-test).

**Supplemental Figure S2**. Additional behavioral and gene expression for wild-type animals treated with Veh or FDP. **(A-C)** Time spent in the Light Zone **(A)**, Entries into the Light Zone **(B)**, and Total Distance Traveled in the Dark-Light Box task **(C)**. *N =* 8 - 11 mice/group. **(D-H)** mRNA expression of *Hmgb1* **(D)**, *Nfkbib* **(E)**, *Nfkbie* **(F)**, *Cry1* **(G)**, and *Nr1d1* **(H)** in the hippocampus and cortex. mRNA expression is compared to the expression of *Hprt* and normalized to the mean of NSD + Veh mice. *N =* 7 - 10 mice / group for hippocampal samples, 5-10 for cortical samples. **p < 0.05,* ***p < 0.01*, ****p<0.001, ****p<0.0001* by Two-Way ANOVA with Tukey’s post-test.

**Supplementary Figure S3. Regulation of IL-1β, Circadian negative arm, and microglia morphology in *Nlrp3^-/-^* mice**. **(A)** Production of IL-1β in the hippocampus and cortex of WT and Nlrp3^-/-^ mice. *N =* 5 - 11 mice / group. **(B)** mRNA expression of circadian clock genes *Cry1* and *Nr1d1* in the hippocampus. *N = 3-9* mice / group. **(C)** Soma volume of CA1 hippocampal microglia. *N =* 4-6 mice / group, > 4 hippocampal sections studied per animal. >25 cells studied per section. **p < 0.05, ***p<0.001, ****p<0.0001* by Two-Way Anova with Tukey’s post-test.

**Supplemental Figure S4. Physiological monitoring data. (A)** Body weight (g), diet consumption (g/day) and liquid consumption of FDP or vehicle (mL/day) in wild-type vehicle-treated mice (WT + Veh), wild-type FDP-treated mice (WT + FDP), *Bmal1*-deficient Vehicle-treated mice (*Bmal1*^-/-^ + Veh), and *Bmal1*-deficient FDP-treated mice (*Bmal1*^-/-^ + FDP) that were tested for Dark-Light Box and Elevated Plus Maze behavior. **(B-E)** Graphic presentation of **(B)** Body weight (g), **(C)** body weight change per week, **(D)** diet consumption (g/day) and **(E)** liquid consumption (mL/day) across the 3-weeks treatment regimen. Data is mean ± SEM (Repeated measures ANOVA with Tukey’s post-test).

**Supplemental Figure S5:** **Regulation of IκB and microglia morphology in *Bmal1^-/-^* mice. (A)** Expression of IκB related genes *Nfkbia*, *Nfkbib*, and *Nfkbie* in the hippocampus. *N =* 3 - 10 mice / group. **(B)** Soma volume of CA1 hippocampal microglia. *N =* 3-4 mice / group, > 4 hippocampal sections studied per animal. >25 cells studied per section. ***p < 0.01, ***p<0.001, ****p<0.0001* by Two-Way Anova with Tukey’s post-test.

**Supplementary Table S1: Polyphenol contents of FDP components.** FDP is comprised of three commercially available components, a select Concord Grape Juice (CGJ), a select Grape Seed Polyphenol Extract (GSPE), and all trans-resveratrol (RSV). In a series of quality control studies, polyphenol contents of CGJ, GSPE, and RSV were analyzed by LC/UV-MS/MS^27^. **(A-C)** Names and concentrations of polyphenols and phenolic acids identified from **(A)** CGJ, **(B)** GSPE, and **(C)** RSV. Phenolic compounds are clustered by their polyphenol structural classes.

**Supplementary Table S2: Biologically available phenolic metabolites from FDP.** Rats were treated orally with FDP. On the last day, rats were given the final dose by gavage followed by collection of plasma. Plasma content of phenolic compounds was analyzed by LC-MS/MS^29,81^. Concentration of polyphenolic compounds from FDP. Phenolic compounds are clustered according to their polyphenol structural classes. For each compound, we present values for plasma concentration and brain concentration. Values are mean ± SD.

**Supplementary Table S3: Biologically available phenolic acids from GSPE.** Rats were treated orally with GSPE. On the last day, rats were given the final dose by gavage followed by collection of plasma. Plasma content of phenolic compounds was analyzed by LC-MS/MS^29,81^. Concentration of polyphenolic compounds from FDP. Phenolic compounds are clustered according to their polyphenol structural classes. For each compound, we present values for plasma concentration and brain concentration. ND: not detectable. Values are mean ± SD.

**Supplementary Table S4: Primers used in this study.** Primers were designed using Primer-BLAST software.
