# Supplementary material for: Anxiolytic effects of NLRP3 inflammasome inhibition in a model of chronic sleep deprivation
Source: Transl Psychiatry. 2021 Jan 14;11:52. doi: 10.1038/s41398-020-01189-3 (PMC7809257; doi:10.1038/s41398-020-01189-3)
Supplement: Supplementary file 2 — Supplementary Figure S1 [file 41398_2020_1189_MOESM2_ESM.pdf]

A

Dark-Light-Box and Elevated Plus Maze	WT NSD + Veh			WT NSD + FDP			WT SD + Veh			WT SD + FDP			*P* (Repeated measure ANOVA)					
													Treatment Effect		Time Effect		Treatment x Time	
	Week 1	Week 2	Week 3	Week 1	Week 2	Week 3	Week 1	Week 2	Week 3	Week 1	Week 2	Week 3	*P*	F (DFn, DFd)	*P*	F (DFn, DFd)	*P*	F (DFn, DFd)
Body Weight (g)	29.22 ± 0.98	29.99 ± 1.21	31.10 ± 1.41	27.07 ± 0.62	27.18 ± 0.62	28.12 ± 0.62	31.28 ± 0.77	31.34 ± 0.84	31.81 ± 1.26	27.66 ±0.88	28.20 ± 0.93	27.80 ± 1.37	0.003	5.68	0.10	2.36	0.80	0.51
Body Weight Change (g)	N/A	0.77 ± 0.49	1.11 ± 0.86	N/A	0.18 ± 0.68	0.93 ± 0.62	N/A	0.06 ± 1.32	0.47 ± 1.37	N/A	0.54 ± 0.27	-0.40 ± 0.65	0.49	0.82	0.84	0.04	0.85	0.27
Diet Consumption (g/day)	3.03 ± 0.82	2.96 ± 0.69	4.19 ± 0.53	2.68 ± 0.37	2.72 ± 0.14	2.97 ± 0.20	3.11 ± 0.52	2.97 ± 0.41	2.53 ± 0.70	2.72 ±0.67	2.75 ± 0.63	2.41± 0.70	0.65	0.59	0.67	0.25	0.31	1.38
Liquid Consumption (mL/day)	3.90 ± 1.27	3.51 ± 0.96	4.28 ± 1.61	3.99 ± 0.19	4.50 ± 0.49	3.94 ± 0.72	3.27 ± 0.68	3.40 ± 0.13	3.13 ± 0.63	5.42 ±1.68	5.68 ± 2.45	2.83 ± 0.93	0.79	0.36	0.26	1.643	0.18	1.86

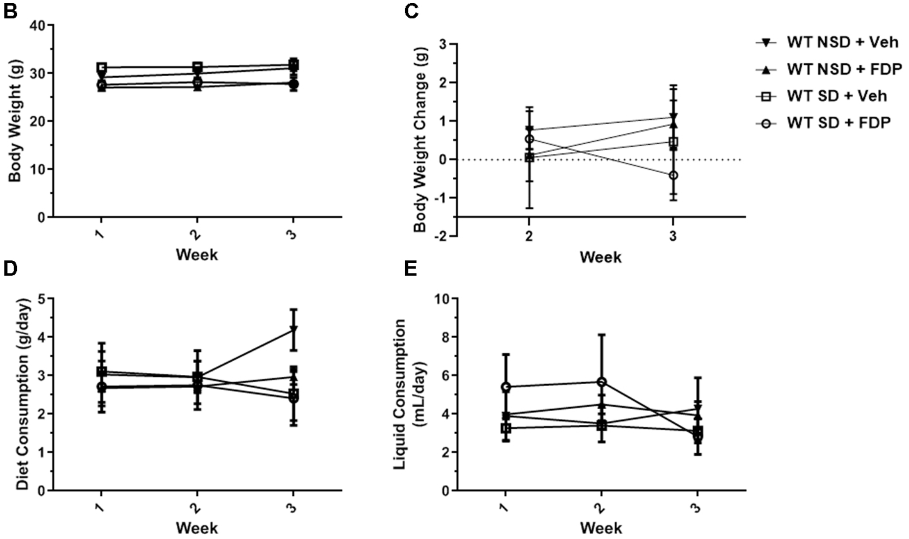

Supplemental Figure S1: Physiological Monitoring Data
