# Supplementary material for: Anxiolytic effects of NLRP3 inflammasome inhibition in a model of chronic sleep deprivation
Source: Transl Psychiatry. 2021 Jan 14;11:52. doi: 10.1038/s41398-020-01189-3 (PMC7809257; doi:10.1038/s41398-020-01189-3)
Supplement: Supplementary file 3 — Supplementary Figure S2 [file 41398_2020_1189_MOESM3_ESM.pdf]

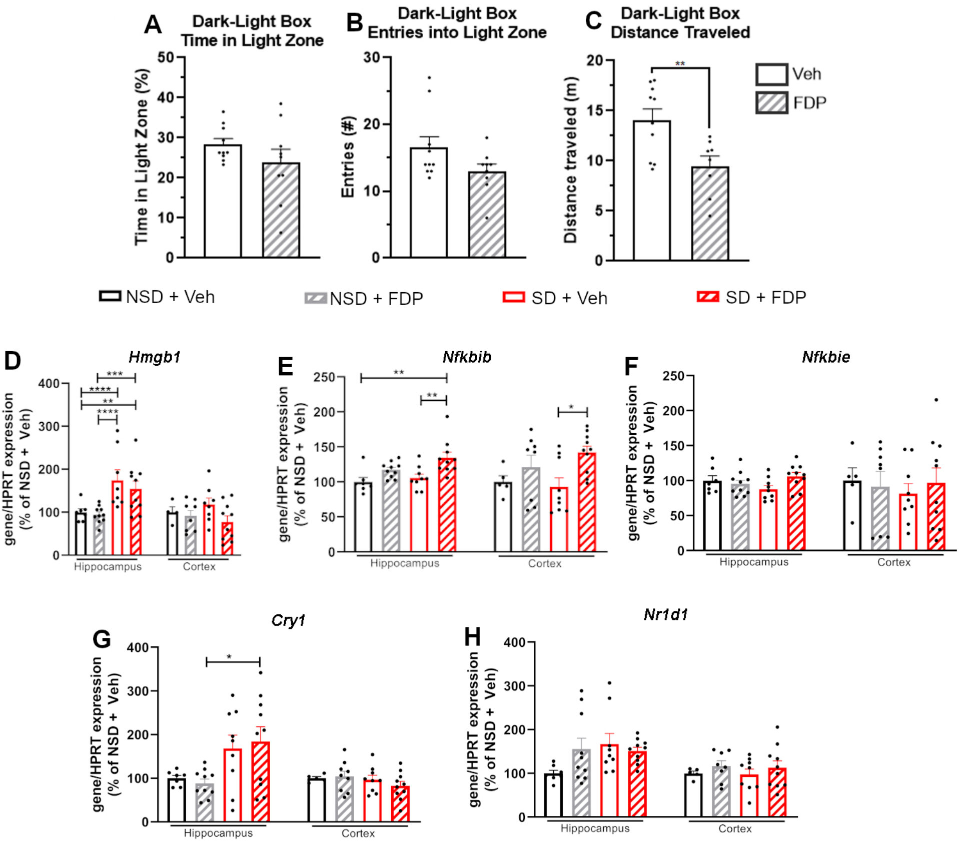

A
Dark-Light Box
Time in Light Zone
Time in Light Zone (%)
B
Dark-Light Box
Entries into Light Zone
Entries (#)
C
Dark-Light Box
Distance Traveled
Distance traveled (m)
**
Veh
FDP
NSD + Veh
NSD + FDP
SD + Veh
SD + FDP
D
Hmgb1
E
Nfkbib
F
Nfkbie
G
Cry1
H
Nr1d1
gene/HPRT expression
(% of NSD + Veh)
Hippocampus
Cortex
***
****
*
