# Supplementary figures and images for: Anxiolytic effects of NLRP3 inflammasome inhibition in a model of chronic sleep deprivation
Source: Transl Psychiatry. 2021 Jan 14;11:52. doi: 10.1038/s41398-020-01189-3 (PMC7809257; doi:10.1038/s41398-020-01189-3)

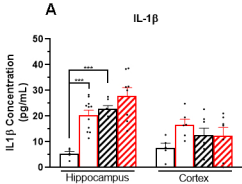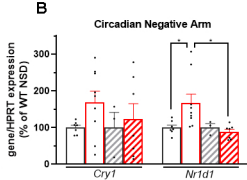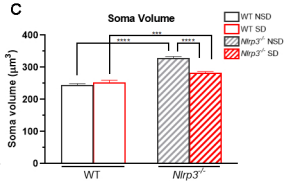

Supplement: Supplementary file 4 — Supplementary Figure S3 [file 41398_2020_1189_MOESM4_ESM.pdf]

**A**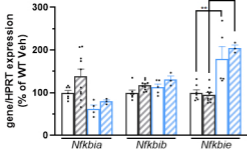**B**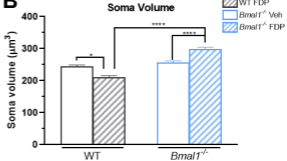

Supplement: Supplementary file 6 — Supplementary Figure S5 [file 41398_2020_1189_MOESM6_ESM.pdf]
