# Supplementary material for: Anxiolytic effects of NLRP3 inflammasome inhibition in a model of chronic sleep deprivation
Source: Transl Psychiatry. 2021 Jan 14;11:52. doi: 10.1038/s41398-020-01189-3 (PMC7809257; doi:10.1038/s41398-020-01189-3)
Supplement: Supplementary file 5 — Supplementary Figure S4 [file 41398_2020_1189_MOESM5_ESM.pdf]

A

Dark-Light-Box and Elevated Plus Maze	WT + Veh			WT + FDP			*Bmal1*$^{-/-}$ + Veh			*Bmal1*$^{-/-}$ + FDP			*P* (Repeated measure ANOVA)					
													Treatment Effect		Time Effect		Treatment x Time	
	Week 1	Week 2	Week 3	Week 1	Week 2	Week 3	Week 1	Week 2	Week 3	Week 1	Week 2	Week 3	*P*	F (DFn, DFd)	*P*	F (DFn, DFd)	*P*	F (DFn, DFd)
Body Weight (g)	29.22 ± 0.98	29.99 ± 1.21	31.10 ± 1.41	27.07 ± 0.62	27.18 ± 0.62	28.12 ± 0.62	24.30 ± 1.57	24.29 ± 1.24	24.85 ± 1.13	20.92 ± 0.69	22.95 ± 0.90	22.93 ± 0.67	<0.0001	13.60	0.0008	8.74	0.34	1.16
Body Weight Change (g)	N/A	0.77 ± 0.49	1.11 ± 0.84	N/A	0.12 ± 0.68	0.93 ± 0.62	N/A	-0.01 ± 0.35	0.57 ± 0.30	N/A	2.03 ± 0.23	-0.02 ± 0.48	0.24	1.48	0.90	0.01	0.41	1.00
Diet Consumption (g/day)	3.03 ± 0.82	2.96 ± 0.69	4.19 ± 0.53	2.68 ± 0.37	2.72 ± 0.14	2.97 ± 0.20	2.64 ± 0.33	2.32 ± 0.01	2.86 ± 0.76	2.13 ± 0.30	2.17 ± 0.03	1.71 ± 0.06	0.06	4.69	0.36	1.06	0.53	0.90
Liquid Consumption (mL/day)	3.90 ± 1.27	3.51 ± 0.96	4.28 ± 1.61	3.99 ± 0.19	4.50 ± 0.49	3.94 ± 0.72	4.68 ± 2.52	2.67 ± 0.17	2.60 ± 0.02	2.54 ± 0.08	4.22 ± 1.33	2.67 ± 0.08	0.71	0.48	0.75	0.28	0.45	1.04

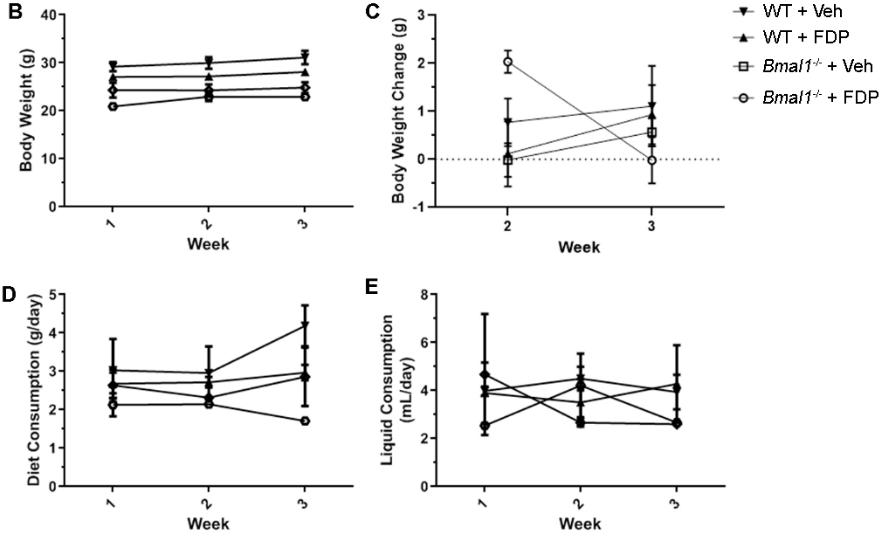

Supplementary Figure S4: Physiological monitoring data.
