# Supplementary material for: Anxiolytic effects of NLRP3 inflammasome inhibition in a model of chronic sleep deprivation
Source: Transl Psychiatry. 2021 Jan 14;11:52. doi: 10.1038/s41398-020-01189-3 (PMC7809257; doi:10.1038/s41398-020-01189-3)
Supplement: Supplementary file 7 — Supplementary Table S1 [file 41398_2020_1189_MOESM7_ESM.docx]

**A.**

| **FDP Component** | **Phenolic compound class**  **Phenolic compound name** | | **Concentration**  **(mg/gm CGJ polyphenol)^*^** |
| --- | --- | --- | --- |
| **Concord Grape Juice (CGJ)** | | | |
|  | **Proanthocyanidins (PA)** | |  |
|  |  | PA-dimers | 59.8 |
|  | **Flavan-3-ols** | |  |
|  |  | Catechin | 45.2 |
|  |  | Epicatechin | 26 |
|  | **Anthocyanidins (Acs)** | |  |
|  |  | Cyanidin-G^**^ | 56.9 |
|  |  | Cyanidin-G-Ac^**^ | 5.4 |
|  |  | Cyanidin-G-Co^**^ | 9.5 |
|  |  | Cyanidin-G-G-Co^**^ | 27.1 |
|  |  | Delphinidin-G^**^ | 74.4 |
|  |  | Delphinidin-G-Ac^**^ | 45.6 |
|  |  | Delphinidin-g-Co^**^ | 21.7 |
|  |  | Malvidin-G^**^ | 16.3 |
|  |  | Malvidin-G-Co^**^ | 3.6 |
|  |  | Malvidin-G-G^**^ | 76.3 |
|  |  | Malvidin-G-G-Co^**^ | 28.9 |
|  |  | Peonidin-G-Co^**^ | 1.2 |
|  |  | Peonidin-G-G-Co^**^ | 13.4 |
|  |  | Petunidin-G-Ac^**^ | 16.1 |
|  |  | Petunidin-G-Co^**^ | 6 |
|  | **Flavonols** | |  |
|  |  | Malvidin-G^**^ | 4.9 |
|  |  | Quercetin-G^**^ | 3.9 |
|  |  | Quercetin-Gln^**^ | 4.2 |
|  |  | Quercetin-3-O-Rutinoside | 5.9 |
|  | **Phenolic Acids** | |  |
|  |  | Gallic Acid | 4.2 |

^*^Unpublished observation

^**^G, glucosyl or galactosyl moiety; Ac, acetyl; Co, coumaroyl

**B.**

| **FDP Component** | **Phenolic compound class**  **Phenolic compound name** | | **Concentration**  **(mg/gm GSPE polyphenol)^*^** |
| --- | --- | --- | --- |
| **Grape Seed Polyphenol Extract (GSPE)** | | | |
|  | **Proanthocyanidins (PA)** | |  |
|  |  | PA-dimers | 79.2 |
|  | **Flavan-3-ols** | |  |
|  |  | Catechin | 33.7 |
|  |  | Epicatechin | 28 |
|  | **Phenolic acids** | |  |
|  |  | Gallic acid | 22.3 |

^*^Unpublished observation

**C.**

| **FDP Component** | **Phenolic compound class**  **Phenolic compound name** | | **Concentration**  **(mg/gm RSV polyphenol)^*^** |
| --- | --- | --- | --- |
| **Resveratrol (RSV)** | | | |
|  | **Stilbenoids** | |  |
|  |  | Resveratrol | 999.9 |

^*^Unpublished observation
