# Supplementary material for: Anxiolytic effects of NLRP3 inflammasome inhibition in a model of chronic sleep deprivation
Source: Transl Psychiatry. 2021 Jan 14;11:52. doi: 10.1038/s41398-020-01189-3 (PMC7809257; doi:10.1038/s41398-020-01189-3)
Supplement: Supplementary file 8 — Supplementary Table S2 [file 41398_2020_1189_MOESM8_ESM.docx]

| **Bioavailability of phenolic metabolites from a Flavonoid-rich Preparation (FDP; 783 mg polyphenol / kg BW / day in plasma and perfused brain specimens from rats)^#^** | | | |
| --- | --- | --- | --- |
| **Polyphenol metabolites** | | **Plasma Concentration (µM)** | **Brain Concentration (pmol/g)** |
| **Flavan-3-ols** | |  |  |
|  | Catechin-5-O-glucuronide | 2.46 ± 0.26 | 485.79 ± 85.07 |
|  | Epicatechin-5-O-glucuronide | 2.79 ± 0.19 | 637.22 ± 93.85 |
|  | 3’0Me-catechin-5-0-glucuronide | 2.82 ± 0.07 | 664.29 ± 133.65 |
|  | 3’0Me-epicatechin-5-0-glucuronide | 4.2 ± 0.13 | 853.83 ± 142.77 |
| **Anthocyanidins** | |  |  |
|  | Cyanidin-glucoside | 0.01 ± 0.001 | 0.07 ± 0.00 |
|  | Delphinidin-glucoside | 0.004 ± 0.0003 | 0.07 ± 0.00 |
|  | Malvidin-glucoside | 0.004 ± 0.0004 | 0.17 ± 0.02 |
|  | Peonidin-glucoside | 0.004 ± 0.0002 | 0.12 ±0.01 |
|  | Petunidin-glucoside | 0.003 ± 0.0005 | 0.10 ± 0.00 |
| **Flavonols** | |  |  |
|  | Quercetin-3-O-glucuronide | 0.11 ± 0.04 | 2.41 ± 0.47 |
|  | Ome-quercetin-O-glucuronide | 0.079 ± 0.008 | 0.69 ± 0.05 |
| **Stilbenoids** | |  |  |
|  | Resveratrol | NA^*^ | NA^*^ |
|  | Resveratrol-3-O-glucuronide | 78.53 ± 3.24 | 746.57 ± 121.73 |

^#^Adapted from Wang et al., Front Aging Neurosci. 6:42, 2014

^*^NA, not assessed
