# Supplementary material for: Anxiolytic effects of NLRP3 inflammasome inhibition in a model of chronic sleep deprivation
Source: Transl Psychiatry. 2021 Jan 14;11:52. doi: 10.1038/s41398-020-01189-3 (PMC7809257; doi:10.1038/s41398-020-01189-3)
Supplement: Supplementary file 9 — Supplementary Table S3 [file 41398_2020_1189_MOESM9_ESM.docx]

| **Bioavailability of phenolic acids from a Grape Seed Polyphenol Extract (GSPE; 250 mg/kg polyphenol BW / day in rat plasma and perfused brain specimens)^#^** | | |
| --- | --- | --- |
| **Phenolic acids** | **Plasma Concentration (µM)** | **Brain Concentration (µM)** |
| ferulic acid | 0.47 ± 0.04 | ND^**^ |
| hippuric acid | 4.72 ± 0.48 | ND^**^ |
| 3-hydroxybenzoic acid | ND^**^ | 1.75 ± 0.30 |
| 4-hydroxybenzoic acid | ND^**^ | ND^**^ |
| 3-hydroxyhippuric acid | ND^**^ | ND^**^ |
| 4-hydroxyhippuric acid | ND^**^ | ND^**^ |
| 3-hydroxyphenylacetic acid | 0.28 ± 0.01 | ND^**^ |
| 3,4-dihydroxyphenylacetic acid | ----- | ND^**^ |
| 3-(3’-hydroxypheyl)propionic acid | 0.92 ± 0.12 | 2.53 ± 0.68 |
| 3-(3’,4’-dihydroxyphenyl)propionic acid | 1.25 ± 0.06 | ND^**^ |
| 5-(4’-hydroxyphenyl)valeric acid | 0.48 ± 0.07 | ND^**^ |
| phenylacetic acid | ND^**^ | ND^**^ |

^#^Wang et al., Mol Nutr Food Res. 59(61):1025,20

^**^ND, not detectable
