# Supplementary material for: Anxiolytic effects of NLRP3 inflammasome inhibition in a model of chronic sleep deprivation
Source: Transl Psychiatry. 2021 Jan 14;11:52. doi: 10.1038/s41398-020-01189-3 (PMC7809257; doi:10.1038/s41398-020-01189-3)
Supplement: Supplementary file 10 — Supplementary Table S4 [file 41398_2020_1189_MOESM10_ESM.docx]

|  | **Primer, 5’ -> 3’** | |
| --- | --- | --- |
| **Gene** | **Forward** | **Reverse** |
| mouse *Hprt* | CCCCAAAATGGTTAAGGTTGC | AACAAAGTCTGGCCTGTATCC |
| mouse *Bmal1* | CACTGTCCCAGGCATTCCA | TTCCTCCGCGATCATTCG |
| mouse *Clock* | GCGACAGCCGGGACACGCCA | CGCGGCGGTAGCGGTGAATTTT |
| mouse *Cry1* | GAGCTCGTGTCCGTTCGTG | CGGAGGACACGCATACCTTC |
| mouse *Cry2* | CCAATCACAGAGGCGATCCA | CGTAGTCCTTTGCGGAACCA |
| mouse *Nr1d1* | CCCCCTGAGCCTTCTTGTAA | GAACAAATCGAGGGGCCAGA |
| mouse *Nlrp3* | GCCATCATCAGCTCCTGTGT | ACTGGCTGACTGAACGACTG |
| mouse *Casp1* | ACTGCTATGGACAAGGCACG | GCAAGACGTGTACGAGTGGT |
| mouse *Il1b* | CTCCATGAGCTTTGTACAAGG | TGCTGATGTACCAGTTGGGG |
| mouse *Hmgb1* | TTCATTTCTCTTTCATAACGAG | TCCAAGAAGTGCTCAGAGAGTG |
| mouse *Nfkbia* | AGGACGAGGAGTACGAGCAA | CGTGATGATTGCCAAGTGC |
| mouse *Nfkbib* | CAGGAGCCAAAACCGACAAC | TGGTGTCAGGTCTGCAATTTT |
| mouse *Nfkbie* | CTCCGGCAAACCACTGCTAT | GATCGGCTCTTCCTCGTCTG |
